# Supplementary material for: The propagation of economic impacts through supply chains: The case of a mega-city lockdown to prevent the spread of COVID-19
Source: PLoS One. 2020 Sep 15;15(9):e0239251. doi: 10.1371/journal.pone.0239251 (PMC7491714; doi:10.1371/journal.pone.0239251)
Supplement: S1 Appendix — (PDF) [file pone.0239251.s001.pdf]

S1 Appendix: Data In the TSR data, the maximum number of suppliers and customers reported by each firm is 24. However, we can capture more than 24 by looking at supplier-customer relations from the opposite direction. Because the TSR data include the address of the headquarters of each firm, we can identify the longitude and latitude of each headquarters by using the geocoding service provided by the Center for Spatial Information Science at the University of Tokyo.

We estimate the value of each transaction between two firms in four steps. First, we divide each supplier's sales into its customers in proportion to the sales of customers, defining a tentative sales value. Second, we transform the industry classifications of the TSR data to those used in the IO table and then aggregate the tentative values at the firm-pair level to obtain the total sales for each pair of sectors computed from the TSR data. Third, we divide the total sales for each sector pair from the TSR data by the transaction values for the corresponding pair in the IO table for Japan in 2015 [23]. Finally, we estimate the transaction values between firms by dividing their transaction values in the TSR data by the aggregate TSR-IO ratio. The final consumption of each sector is allocated to all firms in the sector, using their sales as weights.
