# Supplementary material for: The propagation of economic impacts through supply chains: The case of a mega-city lockdown to prevent the spread of COVID-19
Source: PLoS One. 2020 Sep 15;15(9):e0239251. doi: 10.1371/journal.pone.0239251 (PMC7491714; doi:10.1371/journal.pone.0239251)
Supplement: S2 Appendix — (PDF) [file pone.0239251.s002.pdf]

S2 Appendix: Model We rely on the model of Inoue and Todo [7, 19], an extension of existing agent-based models used to examine the propagation of shocks by natural disasters through supply chains, including Hallegatte's model [20]. Each firm uses a variety of intermediates as inputs and delivers a sector-specific product to other firms and the final consumers. Firms have an inventory of intermediates to address possible supply shortages. S2 Fig provides an overview of the model, showing the flows of products to and from firm i in sector r.

In the initial stage before an economic shock, the daily trade volume from supplier j to customer i is denoted by $A_{i,j}$, whereas the daily trade volume from firm i to the final consumers is denoted as C_i. Then, the initial production of firm i in a day is given by

$$P_{\mathrm{ini}i} = \Sigma_j A_{j,i} + C_i. \tag{1}$$

On day t after the initial stage, the previous day's demand for firm i's product is $D_i^*(t-1)$. The firm thus makes orders to each supplier j so that the amount of its product of supplier j can meet this demand, $A_{i,j} D_i^*(t-1)/P_{\mathrm{ini}i}$. We assume that firm i has an inventory of the intermediate goods produced by firm j on day t, $S_{i,j}(t)$, and aims to restore this inventory to a level equal to a given number of days n_i of the utilization of product of supplier j. The constant n_i is assumed to be Poisson distributed, where its mean is n, which is a parameter. That is, when the actual inventory is smaller than its target, firm i increases its inventory gradually by $1/\tau$ of the gap, so that it reaches the target in τ days, where τ is assumed to be six to follow the original model [20]. Therefore, the order from firm i to its supplier j on day t, denoted as $O_{i,j}(t)$, is given by

$$O_{i,j}(t) = A_{i,j} \frac{D_i^*(t-1)}{P_{\mathrm{ini}i}} + \frac{1}{\tau} \left[n_i A_{i,j} - S_{i,j}(t) \right], \tag{2}$$

where the inventory gap is in brackets. Accordingly, total demand for the product of supplier i on day t, $D_i(t)$, is given by the sum of final demand from final consumers and total orders from customers:

$$D_i(t) = \Sigma_j O_{j,i}(t) + C_i, \tag{3}$$

Now, suppose that an economic shock hits the economy on day 0 and that firm i is directly affected. Subsequently, the proportion $\delta_i(t)$ of the production capital of firm i is malfunctioning. In this paper, δ_i is one during the lockdown if firm i is in Tokyo. Hence, the production capacity of firm i, defined as its maximum production assuming no supply shortages, $P_{\mathrm{cap}i}(t)$, is given by

$$P_{\mathrm{cap}i}(t) = P_{\mathrm{ini}i}(1 - \delta_i(t)). \tag{4}$$

The production of firm i might also be limited by the shortage of supplies. Because we assume that firms in the same sector produce the same product, the shortage of supplies

suffered by firm j in sector s can be compensated for by supplies from firm k in the same sector s. Firms cannot substitute new suppliers for affected suppliers after the disaster, as we assume fixed supply chains. Thus, the total inventory of the products delivered by firms in sector s in firm i on day t is

$$S_{\text{tot}i,s}(t) = \Sigma_{j\in s} S_{i,j}(t). \tag{5}$$

The initial consumption of products in sector s at firm i before the disaster is also defined for convenience:

$$A_{\text{tot}i,s} = \Sigma_{j\in s} A_{i,j}. \tag{6}$$

The maximum possible production of firm i limited by the inventory of product of sector s on day t, $P_{\text{pro}i,s}(t)$, is given by

$$P_{\text{pro}i,s}(t) = \frac{S_{\text{tot}i,s}(t)}{A_{\text{tot}i,s}} P_{\text{ini}i}. \tag{7}$$

Then, we can determine the maximum production of firm i on day t, considering its production capacity, $P_{\text{cap}i}(t)$, and its production constraints due to the shortage of supplies, $P_{\text{pro}i,s}(t)$:

$$P_{\text{max}i}(t) = \text{Min}\left(P_{\text{cap}i}(t), \text{Min}_s(P_{\text{pro}i,s}(t))\right). \tag{8}$$

Therefore, the actual production of firm i on day t is given by

$$P_{\text{act}i}(t) = \text{Min}\left(P_{\text{max}i}(t), D_i(t)\right). \tag{9}$$

When demand for a firm is greater than its production capacity, the firm cannot completely satisfy its demand, as is denoted by Equation (9). In this case, firms should ration their production to their customers. We propose a rationing policy in which customers and final consumers are prioritized if they have small amount of order to their initial order, instead of being treated equally, as in the previous work [20].

Suppose that firm i has customers j and a final consumer. Then the ratio of the order from customers j and the final consumer after the shock to the one before the shock denoted as $O^{rel}_{j,i}$ and O^{rel}_c, respectively, are determined by the following steps, where $O^{sub}_{j,i}$ and O^{sub}_c are temporal variables to calculate the realized order and are set to be zero initially.

1. Obtain the remaining production r of firm i
2. Calculate $O^{rel}_{\text{min}} = \text{Min}(O^{rel}_{j,i}, O^{rel}_c)$
3. If $r \leq (\sum_j O^{rel}_{\text{min}} O_{j,i} + O^{rel}_{\text{min}} C_i)$ then proceed to 8
4. Add O^{rel}_{min} to $O^{sub}_{j,i}$ and O^{sub}_c
5. Subtract $(\sum_j O^{rel}_{\text{min}} O_{j,i} + O^{rel}_{\text{min}} C_i)$ from r
6. Remove the customer or the final consumer that indicated O^{rel}_{min} from the calculation
7. Return to 2
8. Calculate O^{rea} that satisfies $r = (\sum_j O^{rea} O_{j,i} + O^{rea} C_i)$
9. Obtain $O^*_{j,i} = O^{rea} O_{j,i} + O^{sub}_{j,i} O_{j,i}$ and $C^*_i = O^{rea} C_i + O^{sub}_c C_i$, where the realized order from firm j to supplier i is denoted as $O^*_{j,i}(t)$ and the realized order from a final consumer is C^*_i

10. Finalize the calculation

The above rationing is used for the benchmark simulations. In the alternative specification with prioritization within industries, we use the same algorithm, but final consumers are excluded from the calculation. Instead, only when all demand of the customer firms is fulfilled, the remaining production (after rationing customer firms) is assigned to the final consumer.

Under this rationing policy, total realized demand for firm i, $D_i^*(t)$, is given by

$$D_i^*(t) = \Sigma_j O_{i,j}^*(t) + C_i^*, \tag{10}$$

where the realized order from firm i to supplier j is denoted as $O_{i,j}^*(t)$ and that from the final consumers is C_i^*. According to firms' production and procurement activities on day t, the inventory of firm j's product in firm i on day $t+1$ is updated to

$$S_{i,j}(t+1) = S_{i,j}(t) + O_{i,j}^*(t) - A_{i,j} \frac{P_{\mathrm{act}i}(t-1)}{P_{\mathrm{ini}i}}. \tag{11}$$
