# Supplementary material for: The propagation of economic impacts through supply chains: The case of a mega-city lockdown to prevent the spread of COVID-19
Source: PLoS One. 2020 Sep 15;15(9):e0239251. doi: 10.1371/journal.pone.0239251 (PMC7491714; doi:10.1371/journal.pone.0239251)
Supplement: S3 Appendix — (PDF) [file pone.0239251.s003.pdf]

S3 Appendix: Definition of essential industries In this study, the essential industries are excluded from the lockdown. According to the industry classification used in the 2015 national IO table of Japan, we define the essential industries with the codes of 4611, 4621, 4622, 4711, 4811, 5111, 5112, 5711, 5712, 5721, 5722, 5741, 5742, 5743, 5761, 5771, 5781, 5789, 5791, 5911, 5921, 5931, 5941, 5951, 6411, 6421, 6431, and 6441.
