# Supplementary figures and images for: The propagation of economic impacts through supply chains: The case of a mega-city lockdown to prevent the spread of COVID-19
Source: PLoS One. 2020 Sep 15;15(9):e0239251. doi: 10.1371/journal.pone.0239251 (PMC7491714; doi:10.1371/journal.pone.0239251)

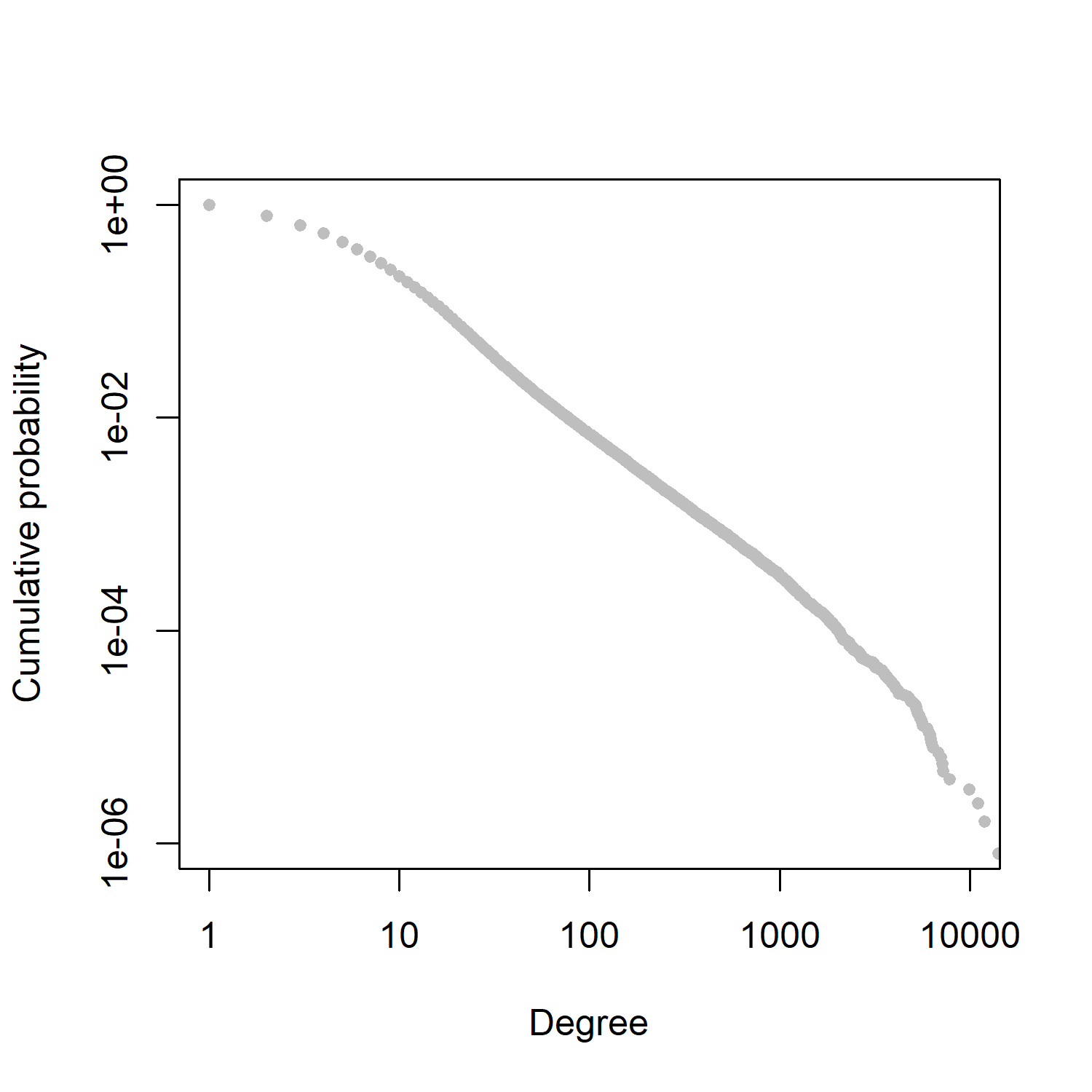

Supplement: S1 Fig — (PNG) [file pone.0239251.s004.png]

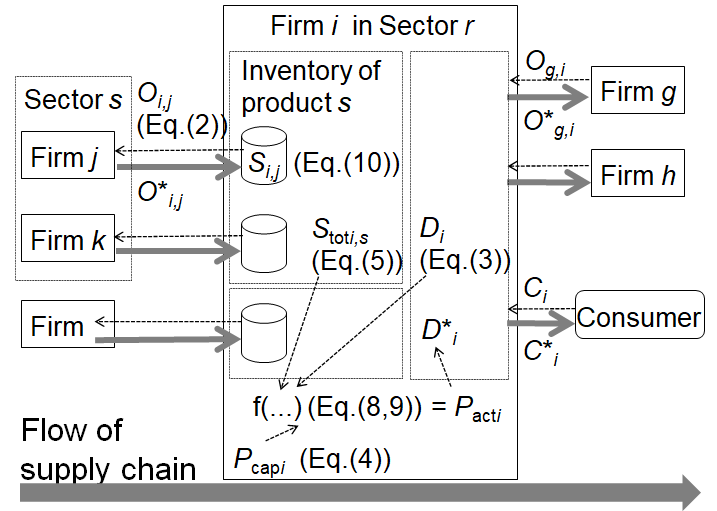

Supplement: S2 Fig — Products flow from left to right, whereas orders flow in the opposite direction. The equation numbers correspond to those in S2 Appendix. (TIF) [file pone.0239251.s005.tif]

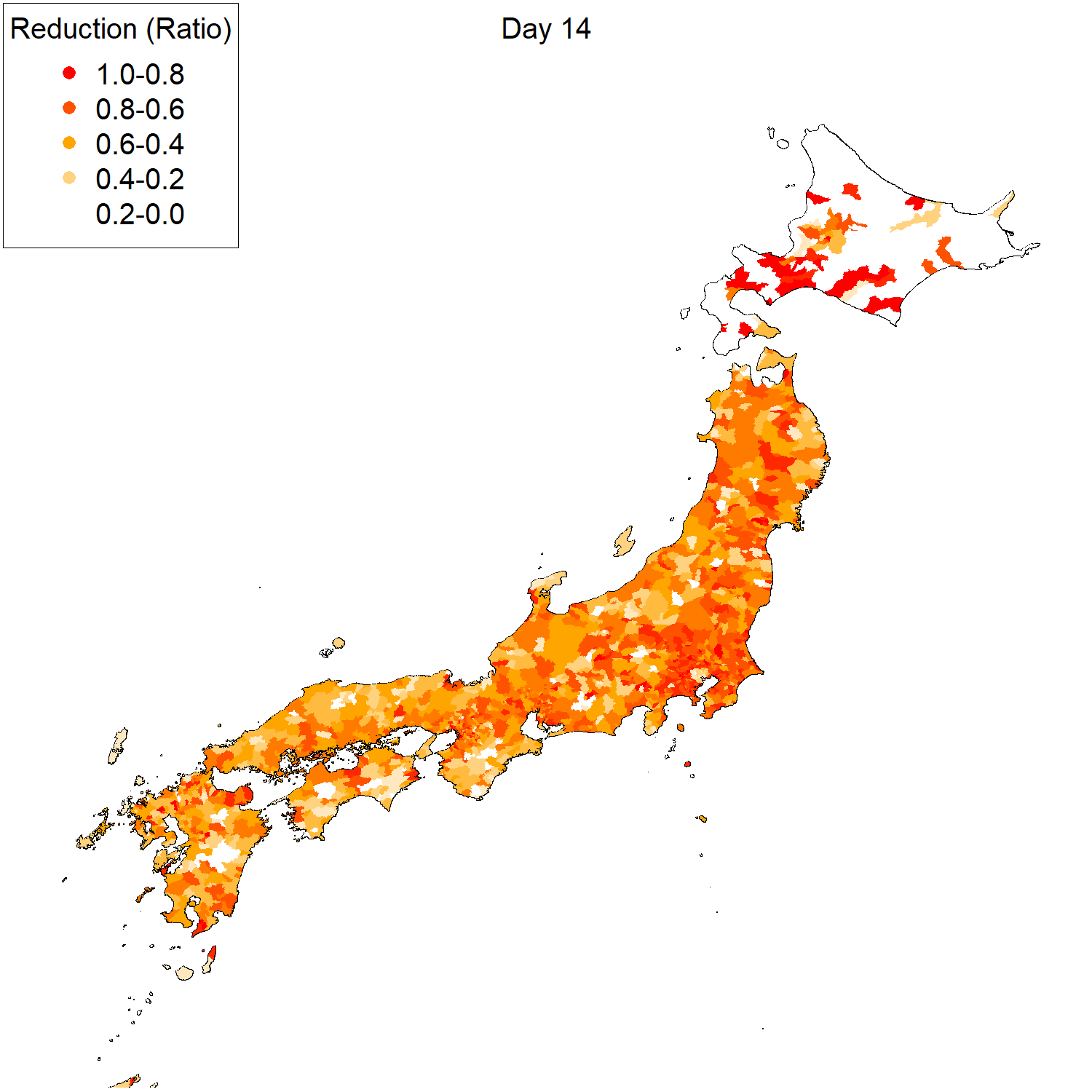

Supplement: S3 Fig — (PNG) [file pone.0239251.s006.png]
