# Supplementary material for: The propagation of economic impacts through supply chains: The case of a mega-city lockdown to prevent the spread of COVID-19
Source: PLoS One. 2020 Sep 15;15(9):e0239251. doi: 10.1371/journal.pone.0239251 (PMC7491714; doi:10.1371/journal.pone.0239251)
Supplement: S1 Table — (Unit: trillion yen). (PDF) [file pone.0239251.s008.pdf]

S1. Table: Results from Alternative Specifications: The loss of value added because of a Tokyo lockdown according to alternative specifications. (Unit: trillion yen)

	Direct effect on Tokyo	Indirect effect on other regions in Japan	Total effect (% of GDP)	Total effect difference with benchmark
A. All production activities are shut down				
1 day	0.471	0.349	0.820 (0.155)	0.259
1 week	3.30	1.56	4.85 (0.916)	1.15
2 weeks	6.60	4.79	11.4 (2.15)	2.13
1 month	14.1	17.0	31.1 (5.89)	3.50
2 months	28.3	43.9	72.1 (13.6)	4.05
B. Producers' use is prioritised over final consumers' use				
1 day	0.309	0.252	0.561 (0.106)	3.32×10^{-4}
1 week	2.17	1.51	3.67 (0.693)	-0.0303
2 weeks	4.33	4.82	9.15 (1.73)	-0.107
1 month	9.28	17.8	27.0 (5.10)	-0.605
2 months	18.6	48.2	66.7 (12.6)	-1.38
C. Proportional shutdown based on the business establishments and factories				
1 day	0.107	0.0749	0.182 (0.0342)	-0.380
1 week	0.747	0.871	1.62 (0.305)	-2.09
2 weeks	1.49	4.19	5.68 (1.07)	-3.58
1 month	3.20	19.3	22.5 (4.25)	-5.10
2 months	6.40	56.2	62.6 (11.8)	-5.52
