# Supplementary material for: The propagation of economic impacts through supply chains: The case of a mega-city lockdown to prevent the spread of COVID-19
Source: PLoS One. 2020 Sep 15;15(9):e0239251. doi: 10.1371/journal.pone.0239251 (PMC7491714; doi:10.1371/journal.pone.0239251)
Supplement: Attachment — Submitted filename: response.pdf [file pone.0239251.s010.pdf]

Response to Editor

1. Please ensure that your manuscript meets PLOS ONE's style requirements, including those for file naming.

   We revised the manuscript and file names to meet the requirements.

2. We note that you have indicated that data from this study are available upon request. PLOS only allows data to be available upon request if there are legal or ethical restrictions on sharing data publicly.

   Technically, what we have declared is not that the data are available upon request but commercially available. As noted in the first submission, the data description is as follows.

   The data for supply chain network are based on a survey done by Tokyo Shoko Research (TSR), one of the leading credit research agencies in Tokyo, supplied to us through the Research Institute of Economy, Trade and Industry (RIETI). The data are not in the public domain but are commercially available.

```
Provider: Tokyo Shoko Research, Ltd.
JA Bldg., 1-3-1 Otemachi, Chiyoda-ku, Tokyo 100-6810, JAPAN
Tel: +81 (0)3-6910-3142
Fax: +81 (0)3-5221-0712
Web: http://www.tsr-net.co.jp/
Database: TSR Company Profile Data File and TSR Business Linkage File
Description: TSR Company Profile Data File is standard data sets that have
been provided for many years in local market. The data is based on TSR's
reporters' site visit interviews which is our most frequent data source.
TSR offers various services/data in accordance with customers' demand,
such as TSR Report, TSR Company Profile Data File, financial statement
data, Internet Service tsrvan2, viability scores, etc.
```

3. We note that Figure 2 in your submission contain map images which may be copyrighted.

   We have used the Global Administrative Areas's shape file for the base file. https://gadm.org/index.html

   The usage permission is open for academic use. https://gadm.org/data.html

4. Please ensure that you refer to Figure 3 in your text as, if accepted, production will need this reference to link the reader to the figure.

   We have clearly indicated that the figure is S1 Fig.

5. We note you have included a table to which you do not refer in the text of your manuscript. Please ensure that you refer to Table 2 in your text; if accepted, production will need this reference to link the reader to the Table.

   We changed the label Table 2 to S1 Table. The S1 Table is referred to in the main text.

Response to Reviewer #1

We cordially thank the reviewer for taking time to review our manuscript. All of the comments are quite helpful for improving the quality of the paper. We have successfully incorporated them in the revised version.

Comment #1-1

The Paper is written in well mannered. In introduction section, author (s) are requested to explain the pinpoint background and the objective of the research. This leads to a greater interest by the reader.

Thank you very much for clarify this shortcoming of the paper. We have taken this comment very seriously and substantially modified the Introduction section, adding paragraphs and several references and reorganizing the structure of the section.

Comment #1-2

Literature review section is not enough to describe the research problem. Author (s) are requested to provide strong literature for present research.

We appreciate the reviewer's comment. We added three recently published studies on the economic effect of COVID-19 and three studies related to network analysis of supply chains in the Introduction section. In the revised version, we use almost one page (page 2) to explain the literature and argue several advantages of our framework compared with those reported in the literature.

Comment #1-3

The conclusions should show the novel results with the past situation research for this authors are advices to include the general findings of the research. It is advisable to provide the limitation of this research in a very concise manner.

Thank you very much for pointing out these important issues. We may have been overly concerned about the effect of COVID-19 and ignored the generalizability of our findings. Therefore, we added the following sentence to emphasize that our conclusion is consistent with the previous literature in the first paragraph of the Discussion and Conclusion section on page 6: "The simulation results clearly show that the effect of a lockdown of Tokyo quickly propagates to other regions outside Tokyo, leading to a substantial effect on the entire Japanese economy. This conclusion is consistent with the previous finding that economic shocks due to natural disasters propagate to non-disaster regions and result in a large total loss [11]."

In addition, we may have been too careful about describing the caveats of this study. Following the reviewer's suggestion, we drastically simplified the caveats in the last part of the main text as follows:

"Several caveats of this study should be mentioned. First, we assume that firms cannot find any new suppliers when supplies from their suppliers in Tokyo are disrupted. Second, for simplicity, the model assumes that even service sectors have the inventory mechanism. Finally, the model in this study only considers the dynamics of production and ignores possible changes in prices and wages incorporated in the literature [23, 24]. These assumptions may be too strong, leading to an overestimation of the propagation effect. However, our main result that the propagation effect is substantial would hold."

Response to Reviewer #2

We cordially thank the reviewer for taking time to review our manuscript. All of the comments are quite helpful for improving the quality of the paper. We have successfully incorporated them in the revised version.

Comment #2-1

As we know, supply chains whether in a country or in the world is a network. Using network quantitative method to explore supply chains is also a major way. This paper employs the dynamic agent-based model of Inoue and Todo that is an extension of the model of Hallegatte, I would like to see the authors spend more time to introduce the differences between the method mentioned in the paper and network quantitative method.

Thank you very much for this important comment. Following this comment, we substantially modified the literature review and motivation part in the Introduction section on pages 2-3. In particular, we added the following paragraph to highlight the difference between our framework and others.

"The analytical framework of Inoue and Todo [11] has several advantages. First, although some studies quantitatively find that supply chains are equipped with complex network characteristics[12-14], they do not examine propagation of shocks through supply chains. Second, the conclusion of Inoue and Todo [11] that the structure of networks significantly influences propagation is consistent with recent findings in the network science literature [15-20]. For example, diffusion is generally promoted when the degree distribution follows a power law and, hence, there are several hub nodes with a large number of links. In such a network, when a shock reaches a hub node, it spreads quickly to many others. Third, although the econometric analysis of [2-5] quantitatively confirms propagation through supply chains, their analysis cannot estimate the total effect of a shock that can be obtained from the framework of Inoue and Todo [11]. Finally, the studies estimating the effect of COVID-19 rely on either a macroeconomic econometric model at the country level [6] or a general equilibrium model assuming international and inter-sectoral IO linkages [7-10], and thus do not incorporate complex inter-firm linkages. As a result, the estimates of the previous studies may be largely undervalued, as suggested by the findings of Inoue and Todo [11]."

Comment #2-2

COVID-19 likes a kind of shock or emergency and many scholars have studied policy shock and public emergency bring about impact on economic or social development, so the author need to show relative literatures published to deeply illustrate the paper's highlights.

We appreciate the reviewer's comment. We added three recently published studies on the economic effect of COVID-19 and describe some of them in detail on page 2 as follows: "For example, McKibbin and Fernando [7]

use a hybrid of dynamic stochastic general equilibrium (DSGE) models and computable general equilibrium (CGE) models assuming international and inter-sectoral input-output (IO) linkages and estimate the effect of the spread of COVID-19 on the world GDP. Guan and others [9] utilize international IO tables at the country-sector level and estimate impacts of lockdowns of heavily infected countries on production in the world economy. According to their estimates, if Europe and the United States imposed a lockdown strategy with a strictness measure of 80% (i.e., 80% of production in most non-essential sectors are shut down) for 2 months, the world value added would decline by 27%."

Comment #2-3

This study attempts to quantify the economic effect of COVID-19 by taking into account the propagation effect across regions through inter-firm supply chains, why does this paper choose firm-level data? I don't quite clear to see the main contributions at the end of the introduction.

This is a very important comment, and we thank the reviewer for raising this issue. To clarify the distinction between the firm-level and region-level analysis and highlight our contribution, we substantially modified the Introduction section and added a paragraph, as we noted above.

Comment #2-4

Please explain further what does Lockdown means and what the actual prerequisites of Lockdown are?

We thank the reviewer for the comment. Following the comment, we added the following paragraph in the Simulation Procedure section:

"It should be noted that our assumption does not exactly match the experience of Japan. In practice, the Japanese government announced the state of emergency for some prefectures including Tokyo from April 7 to May 25, 2020, and for the whole country from April 16 to May 14. According to the Act on Special Measures for Pandemic Influenza and New Infectious Diseases Preparedness and Response, the Japanese government can declare the state of emergency if the spread of a infectious disease, such as COVID-19, is rapid and nationwide. In the state of emergency, some economic activities were requested to be closed, and people were requested to reduce their contacts with others by 80%. Although there was no legal punishment for disobeying the requests, strong social pressure in Japan led people and businesses to voluntarily restrict their activities to a large extent. As a result, production activities including those in sectors not officially restricted were reported to shrink substantially (Mainichi Newspaper, May 27, 2020). Because it is still unclear to what extent economic activities shrank, we assume a complete shutdown of non-essential production activities. In addition, to highlight the propagation of the effect of a lockdown of a mega-city to other regions, we focus on a lockdown of Tokyo rather than the entire

economy."

Comment #2-5

I would like to see the process of how to match firm-level data with IO industry-level data?

We apologize for not providing enough information about the process. Following this comment, we modified the second paragraph of S1 Appendix as follows:

"We estimate the value of each transaction between two firms in four steps. First, we divide each supplier's sales into its customers in proportion to the sales of customers, defining a tentative sales value. Second, we transform the industry classifications of the TSR data to those used in the IO table and then aggregate the tentative values at the firm-pair level to obtain the total sales for each pair of sectors computed from the TSR data. Third, we divide the total sales for each sector pair from the TSR data by the transaction values for the corresponding pair in the IO table for Japan in 2015 [25]. Finally, we estimate the transaction values between firms by dividing their transaction values in the TSR data by the aggregate TSR-IO ratio. The final consumption of each sector is allocated to all firms in the sector using their sales as weights."
