# Supplementary material for: The propagation of economic impacts through supply chains: The case of a mega-city lockdown to prevent the spread of COVID-19
Source: PLoS One. 2020 Sep 15;15(9):e0239251. doi: 10.1371/journal.pone.0239251 (PMC7491714; doi:10.1371/journal.pone.0239251)
Supplement: Attachment — Submitted filename: Comment_POS.docx [file pone.0239251.s009.docx]

The Paper is written in well mannered. In introduction section, author (s) are requested to explain the pinpoint background and the objective of the research. This leads to a greater interest by the reader.

Literature review section is not enough to describe the research problem. Author (s) are requested to provide strong literature for present research.

The conclusions should show the novel results with the past situation research for this authors are advices to include the general findings of the research. It is advisable to provide the limitation of this research in a very concise manner.

The overall work done by the author is good. I recommend this paper for acceptance with minor revision as suggested above.
